# After an initial balance favoring collagen formation and mineralization, bone turnover markers return to pre-treatment levels during long-term TNF-α inhibition in patients with ankylosing spondylitis

**DOI:** 10.1371/journal.pone.0283579

**Published:** 2023-03-24

**Authors:** Mark Siderius, Anneke Spoorenberg, Frans G. M. Kroese, Eveline van der Veer, Suzanne Arends

**Affiliations:** 1 Rheumatology and Clinical Immunology, University Medical Center Groningen, University of Groningen, Groningen, The Netherlands; 2 Laboratory Medicine, University Medical Center Groningen, University of Groningen, Groningen, The Netherlands; Ohio State University, UNITED STATES

## Abstract

**Background:**

Bone turnover balance favors bone formation, especially mineralization, during the first 3 years of treatment with TNF-α inhibitors (TNFi). Our aim was to evaluate the course of serum bone turnover markers (BTM) and to investigate if facilitation of mineralization reflected by BTM BALP continues to increase during 6 years of TNFi treatment in patients with ankylosing spondylitis (AS) in daily clinical practice.

**Methods:**

Included were outpatients from the University Medical Center Groningen (UMCG) participating in the Groningen Leeuwarden Axial SpA (GLAS) cohort who were treated with TNFi for at least 6 years. Serum markers of collagen resorption, bone regulation, collagen formation and facilitator of bone mineralization (sCTX, OC, PINP and BALP, respectively) were measured at baseline, 3 and 6 months, 1, 2, 4 and 6 years. Z-scores were calculated to correct for age and gender.

**Results:**

53 AS patients were eligible for analyses (66% male, mean age 39±11 years). Disease activity showed rapid and sustained improvement after start of TNFi. Evaluating BTM, sCTX did not significantly change during 6 years of treatment. OC was only significantly increased at 3 months compared to baseline, with median change in Z-score of +0.5. PINP significantly increased at 3 and 6 months and 2 years of treatment, with maximum median change in Z-score of +0.3. Interestingly, BALP was significantly increased at all time points up to and including 2 years of TNFi treatment, with maximum change in median Z-score of +1.2, and decreased thereafter.

**Conclusion:**

In AS patients receiving long-term TNFi, bone turnover balance favored collagen formation and facilitation of mineralization during the first 2 years of treatment. Thereafter, at 4 and 6 years of follow-up, BTM Z-scores returned to pre-treatment levels.

## Introduction

Ankylosing spondylitis (AS), a chronic auto-inflammatory disease of especially the sacroiliac joints (SI) and spinal column, is characterized by inflammation, osteoproliferation and excessive bone loss [1]. Serum levels of bone turnover markers (BTM) are valuable to elucidate bone metabolism during the disease process in AS. However, in AS patients, there seems a dual effect of the disease on bone metabolism, resulting into both excessive bone formation (e.g. syndesmophytes and spinal ankylosis) and bone loss (e.g. osteoporosis and vertebral fractures) makes interpretation more complex [2].

In AS patients with persisting high disease activity despite conventional therapy, switch to biologicals and tumor necrosis factor-alpha (TNF-α) inhibitors (TNFi) to start as a first biological is advised [3]. Inhibition of TNF-α has proven to reduce systemic inflammation on the long-term [4]. TNF-α signaling is an important contributor to pathogenic inflammatory bone metabolism by inducing osteoclast formation and inhibiting collagen formation by osteoblasts. Accordingly, TNFi directly and indirectly influences bone metabolism reflected by changes in BTM and also bone mineral density (BMD) [1]. BTM may not only mirror the effect of disease activity, but are also associated with disease outcome such as the presence of spinal ankylosis as well as low BMD in AS [1].

In a placebo-controlled trial of 201 axSpA patients treated with infliximab for 2 years, baseline levels of collagen resorption marker serum type I collagen C-telopeptide (sCTX), bone regulation marker osteocalcin (OC) and facilitator of bone mineralization marker bone-specific alkaline phosphatase (BALP) were positively correlated with an increase in spinal BMD at 24 and 102 weeks of follow-up [5]. In our previous prospective cohort study of 72 AS patients treated with TNFi for 3 years, we found that bone turnover balance favored bone formation, especially mineralization [6]. Excessive mineralization of bone may also increase the risk of fractures [7]. This raises the question if more long-term TNFi treatment leads to prolonged mineralization reflected by BTM.

Therefore, our goal was to evaluate the course of serum bone turnover markers (BTM) and to investigate if the facilitation of mineralization reflected by BTM BALP still continues to increase during 6 years of TNFi treatment in patients with AS in daily clinical practice.

## Methods

### Patients

Included were 53 consecutive AS outpatients from the University Medical Center Groningen (UMCG) participating in the Groningen Leeuwarden Axial SpA (GLAS) cohort who were treated with TNFi for at least 6 years, with a maximum of 2 different TNFi. Patients were excluded from analyses when they used antiresorptive drugs to treat osteoporosis (e.g. bisphosphonates) at baseline or during follow-up. Data for a specific visit was coded as missing when patients either had experienced a fracture or received systemic corticosteroids within 1 year from that particular visit due to the possible effect on BTM. All patients were over 18 years of age, fulfilled the modified New York criteria for AS and/or the Assessments in Ankylosing Spondylitis (ASAS) criteria for AS including MRI and started TNFi because of active disease according to the ASAS consensus statement [3, 7, 8]. Etanercept was administered as a subcutaneous injection once (50 mg) or twice (25 mg) a week. Infliximab (5 mg/kg) was given intravenously at 0, 2 and 6 weeks and then every 8 weeks. In case of inadequate response, the frequency of infliximab treatment was raised to every 6 weeks. Adalimumab (40 mg) was administered as a subcutaneous injection on alternate weeks. If needed, dosage levels or frequency was increased or decreased based on therapy response, side effects or co-morbidity. Switching to a second TNFi could occur during follow-up, i.e. because of intolerance due to adverse events or inefficacy. The GLAS cohort was approved by the local ethics committees of the UMCG and the Medical Center Leeuwarden. All patients provided written informed consent according to the Declaration of Helsinki.

### Clinical assessments

Demographics characteristics and clinical assessments were obtained from the regular GLAS outpatient visits: age, gender, symptom duration, human leukocyte antigen-B27 (HLA-B27) status, smoking status, history of extra-skeletal manifestations (inflammatory bowel disease (IBD), uveitis or psoriasis), history of peripheral arthritis, and medication use. Disease activity was assessed with AS Disease Activity Score with CRP (ASDAS_CRP_), Bath AS Disease Activity Index (BASDAI) and C-reactive protein (CRP). Physical function was assessed using spinal mobility assessments including occiput to wall distance, chest expansion, modified Schober test, and lateral lumbar flexion. Serum levels of 25-hydroxyvitamin D (25OHvitD) were measured by radioimmunoassay (RIA; DiaSorin, Stillwater, MN, USA; until June 2010) or automated LC-MS method (since 2010).

### BMD measurements

BMD of the lumbar spine (anterior-posterior projection L1-L4) was measured at baseline, 2, 4 and 6 years using dual-energy X-ray absorptiometry (Hologic QDR Discovery, Waltman, MA, USA). Z-scores, the number of SD from the normal mean corrected for age and gender, were calculated using the NHANES reference database. Low BMD was defined as lumbar spine Z-score ≤1.

### Spinal radiographic outcome

Available radiographs of the cervical and lumbar spine were scored at baseline, 2, 4 and 6 years in chronological time order by two trained readers blinded for patient characteristics using the modified Stoke AS Spine Score (mSASSS; range 0–72). Longitudinal radiographs of included patients treated with TNFi were randomized and scored together with longitudinal radiographs of axSpA patients not treated with TNFi in order to avoid potential reader bias concerning the effect of treatment on mSASSS. The average mSASSS total score of the two readers was used in the analysis. When there was discrepancy of >5 points in mSASSS total scores, a third independent reader scored the radiographs [9–11].

### Bone turnover markers

Standardized follow-up visits were performed at baseline (before start of TNFi), 3 and 6 months, 1, 2, 4 and 6 years. Bone turnover was studied by assessment of serum markers of bone resorption (sCTX), bone regulation (OC), collagen formation (procollagen type 1 N-terminal peptide; PINP) and facilitator of bone mineralization (BALP). sCTX was measured by electro-chemiluminescence (ECLIA; Elecsys 2010 Roche Mannheim, Germany; inter-assay coefficient of variation (IE-CV) 10.8%), OC by immunoradiometric assay (IRMA; BioSource Europe South Africa; IE-CV 9.4%), PINP by RIA (Orion Diagnostica, Espoo, Finland; IE-CV 9.0%) and BALP by enzyme-linked immunosorbent assay (ELISA; Metra Biosystems, Mountain View, CA, USA; IE-CV 5.5%). Bone turnover markers were measured in a NEN-EN-ISO 9001:2008 certified and NEN-EN-ISO 15189:2012 accredited laboratory. Serum was acquired of non-fasting patients during study visits of the GLAS cohort taking place at fixed hours (same half-day) and were stored within one hour at -20°C until analysis. BTM Z-scores were used for analyses. Z-scores express the number of standard deviations (SD) from the normal mean corrected for age and gender, and therefore were used to correct for the normal influence that age and gender have on bone turnover. These BTM Z-scores were calculated using matched 10-year-cohorts of a Dutch reference group (200 men or 350 women), checked for serum 25OHvitD levels >50 nmol/liter as well as for the absence of osteoporosis (BMD T-score >-2.5) after 50 years of age: (BTM value of individual patient–mean BTM value of matched 10-year-cohort of reference group) / SD of matched reference cohort. Additionally, the net effect of collagen metabolism was explored by calculating: collagen formation marker PINP Z-score–collagen degradation marker sCTX Z-score.

### Statistical analysis

Statistical analysis were performed with PASW Statistics 23 (SPSS, Chicago, IL, USA). Results were expressed as mean ± SD or median (interquartile range P25-P75) for normally distributed and non-normally distributed data, respectively. Generalized estimating equations (GEE) were used to analyze BTM, BMD, and clinical assessments over time within subjects. GEE is a longitudinal analysis technique which makes use of all available longitudinal data and allows unequal numbers of repeated measurements [12]. Missing data were not imputed. In case residuals were non-normally distributed, variables were transformed (log or square root) before being entered into the model. Different correlation structures (exchangeable, M-dependent, unstructured) were tested and the model with the best fit for the data based on the lowest corrected quasi-likelihood information criterion was used. GEE were corrected for serum levels of 25OHvitD over time. Simple contrasts were used to compare baseline and follow-up visits. P values <0.05 were considered statistically significant.

## Results

### Study population

**Fig 1:**
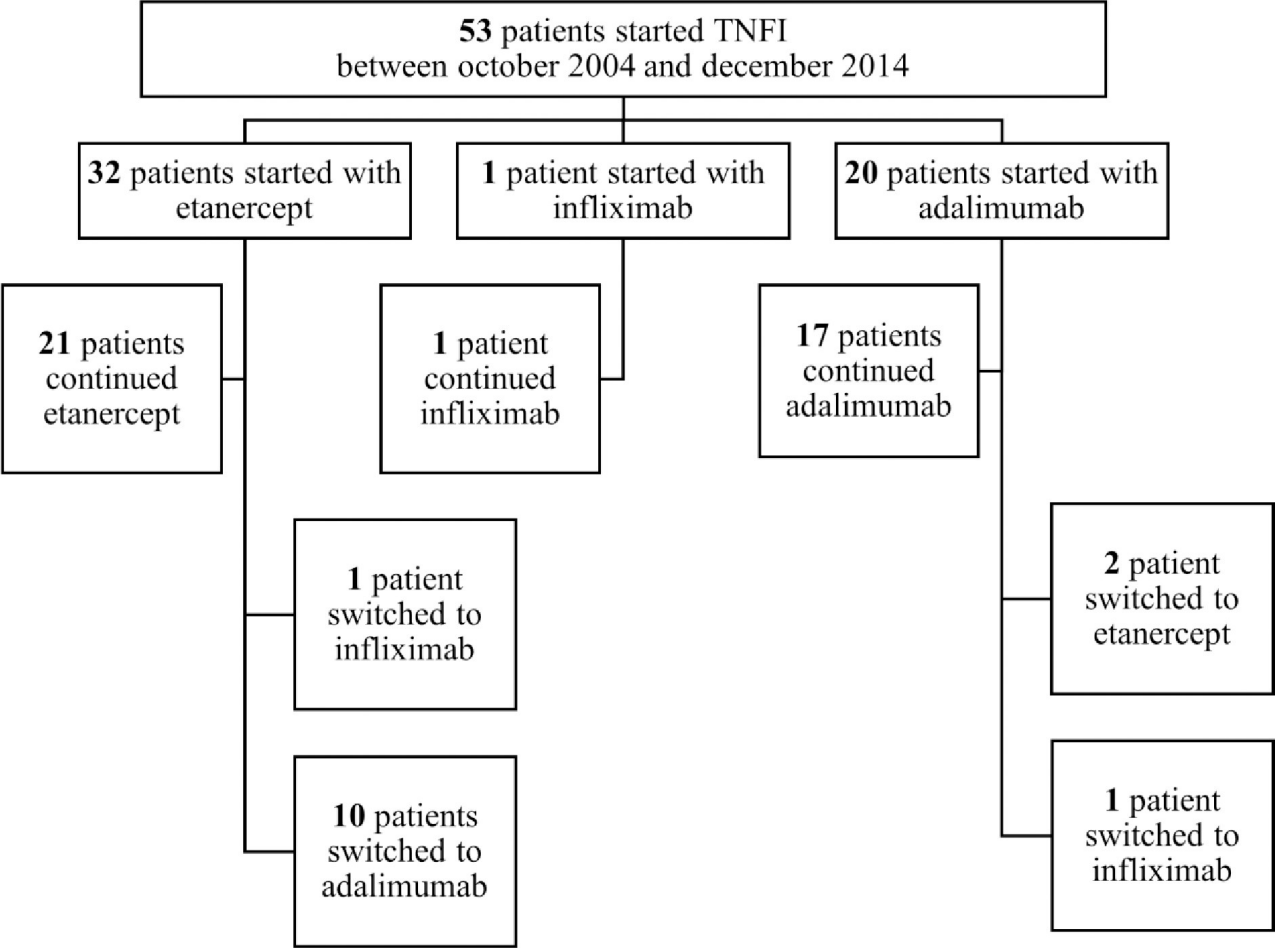
Flowchart of TNFi use of the 53 AS patients on TNFi for ≥6 years and ≤ 2 different TNFi.

In total, 53 AS patients started TNFi between October 2004 and December 2014 and had a follow-up of at least 6 years. Baseline characteristics showed that 66% were male, mean age was 38.5 ± 11.3 years, median symptom duration 15 years (IQR 9–25), 87% were HLA-B27+, mean ASDAS was 3.8 ± 1.0, mean BASDAI 5.7 ± 2.0 and median CRP 14 mg/L (IQR 7–27) (Table 1). At baseline, low BMD at the lumbar spine was present in 36% of the patients. mSASSS was available in 33 of 53 included AS patients, with median 2.7 (IQR 0.8–10.1) and mean 7.3 ± 10.3. Etanercept, infliximab or adalimumab was prescribed as first TNFi in 60%, 2% and 38% of patients, respectively. 26% (n = 14) of patients switched to a second TNF-α inhibitor during follow-up (Fig 1). Reasons for switching were infections (n = 6), secondary inefficacy in combination with infections (n = 2), cardio-vascular symptoms (n = 2), allergic skin reaction (n = 1), development of inflammatory bowel disease (n = 1) or miscellaneous (n = 2) (Fig 1).

**Table 1:** Baseline characteristics of the AS study population treated with TNFi for 6 years (n = 53).

Age (yrs)	38.5 ± 11.3
Gender (male) (n, %)	35 (66)
Duration of symptoms (yrs)	15 (9–25)
HLA-B27^+^ (n, %)	46 (87)
History of IBD (n, %)	4 (8)
History of uveitis (n, %)	11 (22)
History of psoriasis (n, %)	1 (2)
History of peripheral arthritis (n, %)	15 (31)
Current NSAID use (n, %)	46 (90)
Current DMARD use (n, %)	13 (25)
BASDAI (range 0–10)	5.7 ± 2.0
BASDAI ≥4 (n, %)	43 (84)
ASDAS_CRP_	3.8 ± 1.0
ASDAS_CRP_ ≥2.1 (n, %)	47 (94)
CRP (mg/L)	14 (7–27)
Increased CRP >5 (n, %)	43 (84)
Occiput to wall distance (cm)	0.0 (0.0–8.0)
Chest expansion (cm)	3.0 (2.0–4.0)
Modified Schober test (cm)	3.1 ± 1.4
Lateral lumbar flexion mean (cm)	10.2 ± 5.6
25(OH)D (nmol/L)	60.9 ± 26.5
sCTX (pg/mL)	254.8 ± 111.1
sCTX Z-score	0.8 ± 1.0
Osteocalcin (ng/mL)	12.1 ± 4.3
Osteocalcin Z-score	-0.5 ± 0.7
PINP (μg/L)	48.7 ± 18.4
PINP Z-score	0.3 ± 0.9
BALP (U/L)	21.3 ± 7.4
BALP Z-score	1.1 ± 1.9
BMD LS Z-score	-0.3 (-1.4–0.7)
BMD Z≤-1 (n, %)	16 (36)
mSASSS	2.7 (0.8–10.1)

Values are mean ± SD or median (range) unless otherwise indicated. AS: Ankylosing Spondylitis; HLA-B27+: human leukocyte antigen B27 positive; IBD: inflammatory bowel disease; NSAID: non-steroidal anti-inflammatory drug; DMARD: disease-modifying antirheumatic drug; BASDAI: Bath Ankylosing Spondylitis Disease Activity Index; ASDAScrp: ankylosing spondylitis disease activity score with CRP; CRP: C-reactive protein; BALP: bone-specific-alkaline phosphatase; PINP: procollagen type 1 N-terminal peptide; sCTX: serum C-telopeptide of type I collagen; BMD: bone mineral density; LS: lumbar spine; mSASSS: modified Stoke ankylosing spondylitis spinal score.

### Disease activity during 6 years of TNFi

**Fig 2:**
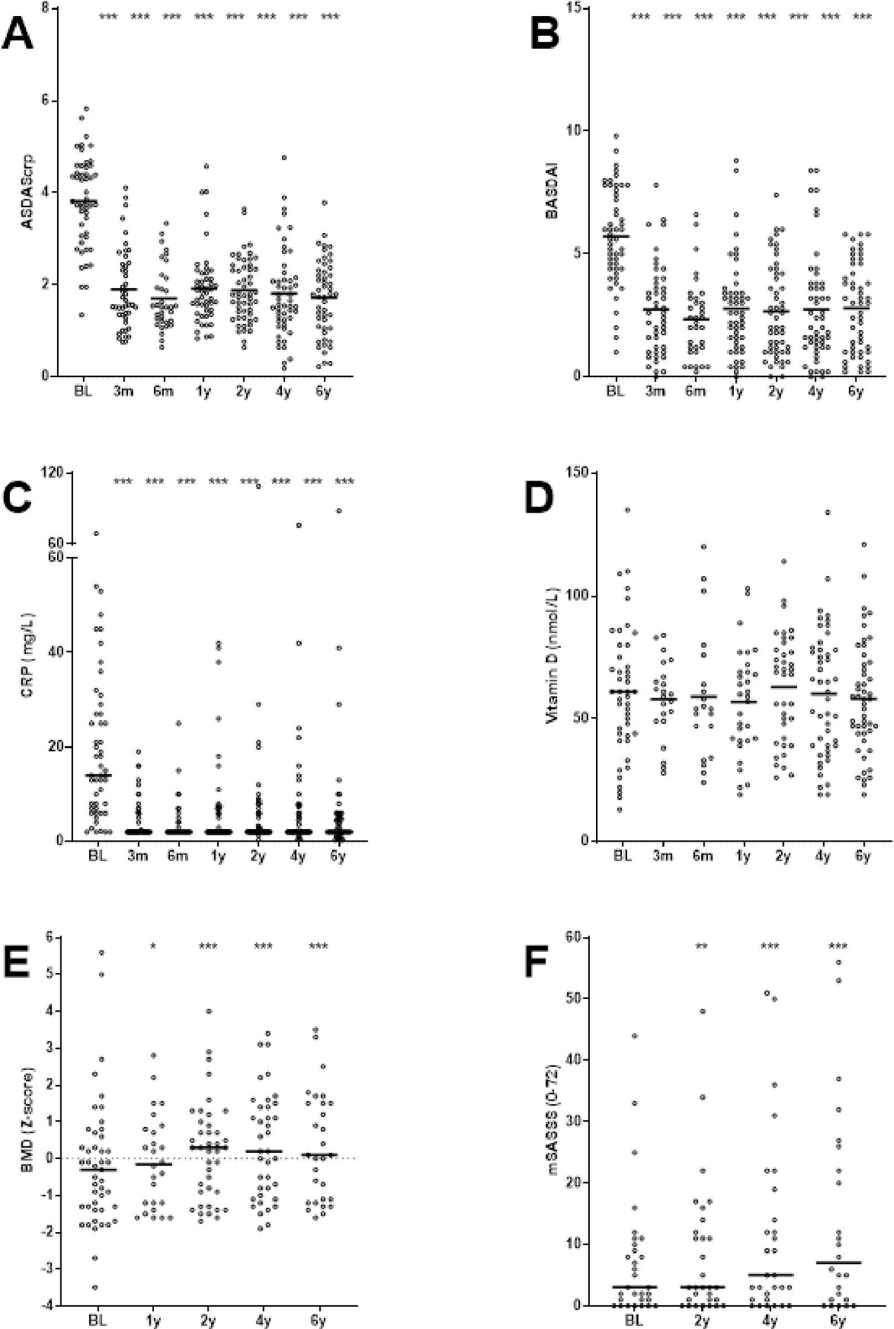
Disease activity index score ASDAScrp (A), BASDAI (B), levels of acute phase reactant CRP (C), levels of vitamin D (D), BMD (E) and mSASSS progression (F) during 6 years of treatment with TNFi in patients with AS (n = 53). * indicate p-value of <0.05, ** indicate p-value of <0.01 and *** indicate p-value of <0.001 compared to baseline. Horizontal lines represent mean (Fig A, B and D) or median (C, E and F) and dots represent individual values.

After start of TNFi, all disease activity assessments showed a rapid and sustained improvement. Both ASDAScrp and BASDAI decreased significantly during TNFi at all follow-up visits compared to baseline (Fig 2). Mean change in ASDAScrp was 2.0 ± 1.0 at 6 years compared to baseline. Furthermore, 81% of patients were clinical responder based on improvement in ASDAScrp of >1.1 at 6 years. Levels of acute phase reactant CRP quickly decreased following therapy and were found to remain significantly lower compared to baseline. However, CRP levels remained elevated (>5mg/L; mean 16 mg/L) in approximately 10% of patients at every visit during follow-up. Serum levels of 25OHvitD were stable at group level during follow-up, although 8% and 28% of the patients had baseline 25OHvitD <25 or <50 nmol/L, respectively. BMD Z-score of the lumbar spine improved significantly during TNFi at all follow-up visits compared to baseline. Significant improvement compared to the previous time point was found up to and including 4 years, thereafter, flattening of improvement was observed. Mean increase in mSASSS was 1.6, 1.3 and 1.2 points for 0–2, 2–4 or 4–6 years, respectively (Fig 2).

### Bone turnover markers during 6 years of TNFi

**Fig 3:**
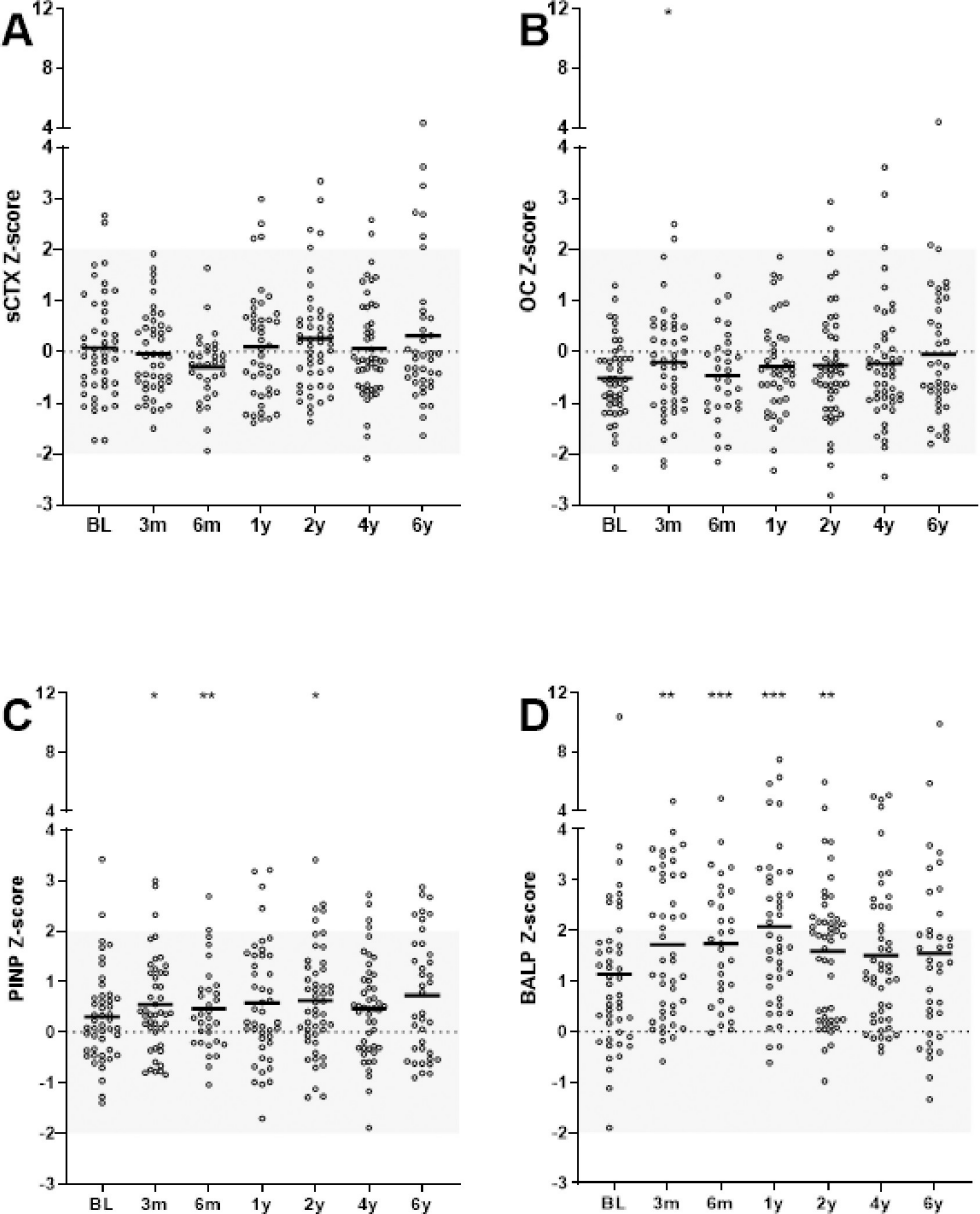
The effect of 6 years of TNFi on markers of bone turnover sCTX (A), OC (B), PINP (C) and BALP (D) in patients with AS (n = 53). * indicate p-value of <0.05, ** indicate p-value of <0.01 and *** indicate p-value of <0.001 compared to baseline. Horizontal lines represent mean and dots represent individual values.

**Fig 4:**
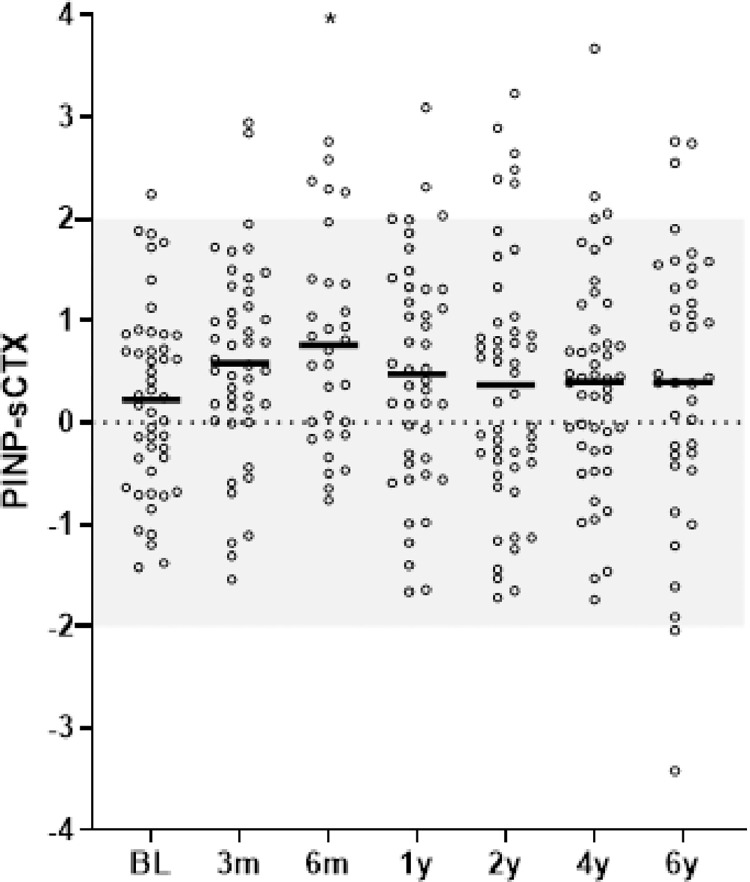
The net effect of collagen formation and degradation during 6 years of TNFi. * indicate p-value of <0.05 compared to baseline. Horizontal lines represent mean and dots represent individual values.

At baseline, median BTM Z-scores were -0.08, -0.55, 0.14 and 0.83 for sCTX, OC, PINP and BALP, respectively. At group level, bone resorption marker sCTX did not significantly change over time after start of TNFi. Bone regulation marker OC was significantly increased after 3 months of TNFi compared to baseline, with a median change in Z-score of +0.5. Bone collagen formation marker PINP was significantly increased at 3 and 6 months and 2 years of TNFi compared to baseline, with a maximum median change in Z-score of +0.3. Facilitator of bone mineralization marker BALP was significantly increased at all time points up to and including 2 years compared to baseline, with a maximum median change in Z-score of +1.2. Thereafter, at 4 and 6 years, BALP returned to levels not significantly different from baseline (Fig 3A–3D). All results remained similar when correcting for serum levels of 25OHvitD over time. Exploring the net effect of collagen metabolism (collagen formation marker PINP Z-score–collagen degradation marker sCTX Z-score) confirmed that the balance favored collagen formation. However, a significant increase in the net effect of collagen formation was only observed between baseline and 6 months with a median PINP–sCTX Z-score of 0.25 and 0.71 at baseline and 6 months, respectively (Fig 4). The number of patients with a BALP Z-scores above +2 SD was highest after 1 and 2 of TNFi treatment; in 22%, 45%, 41%, 28% and 27% of the patients at baseline, 1, 2, 4 or 6 years, respectively.

## Discussion

This prospective study within the observational GLAS cohort is the first study that evaluated the prolonged course of BTM during continuous TNFi treatment in AS patients in daily clinical practice. At group level, the collagen degradation and formation products indicated a balance towards bone formation, especially mineralization, in the first 2 years of TNFi treatment, which is in concordance with our previous study of AS patients with 3 years of TNFi treatment [6]. In addition, the present study showed that OC, a regulating factor in the process of bone formation, was only elevated in the first months after start of TNFi. Most interestingly, BALP, an essential facilitator of bone mineralization, remained elevated for 2 years and decreased thereafter. Therefore, the course of BTM serum levels over time during long-term treatment with TNFi seems to reflect the process of radiographic damage [13, 14]. Besides radiographic bone formation, secondary osteoporosis, reflected by low BMD especially at the lumbar spine can frequently be observed in AS patients. In this study, low BMD at the lumber spine was found in 36% of the patients at baseline. In our previous cross-sectional analysis of AS patients with active disease, higher serum levels of sCTX, and to a lesser extent PINP, were associated with the presence of complete bridging. Higher sCTX Z-scores were also found to be significantly associated with low BMD [15].

Mineralization, of which the outcome is BMD, from a physiological point of view is the enlargement of hydroxyapatite crystals with displacement of water from the collagen fibrils and fibers. This is a slow process which takes months, in which BALP is an essential facilitator. Previous research in AS demonstrated that following TNFi BMD of the lumbar spine (AP projection) significantly improves within 24 weeks [5, 6]. Serum levels of BTM resemble this course in BMD. Also in this study, we observed a persistent improvement in BMD of the lumbar spine compared to baseline accompanied by a BTM balance that initially favors bone formation. Significant improvement compared to the previous timepoint was observed up to and including 4 years. Noteworthy, this long-term course of improvement in BMD coheres with the course of BTM BALP.

The pro-inflammatory cytokine TNF-α plays an important role in the inflammatory process of AS and particularly impacts bone metabolism. In healthy individuals, the process of bone metabolism, i.e. osteoclastogenesis and osteoblastogenesis, are well balanced ensuring the continued remodeling and renewing of the skeleton [16, 17]. TNF-α signaling is an important contributor to pathogenic inflammatory bone metabolism by inducing osteoclast formation and inhibiting collagen formation by osteoblasts [18, 19]. It is well established that TNF-α induces differentiation of osteoclast precursors present in the bone marrow macrophage population and thus stimulates osteoclastogenesis. It is unclear whether TNF-α is able to induce differentiation from precursors alone or needs synergy from other cytokines as IL-1α [19]. Upon initiation of TNFi, in our study, a slight but non-significant decrease was observed in sCTX during the first 6 months. Previously, we found that an early decrease of sCTX during the first 3 months after starting TNFi is inversely predictive for treatment discontinuation [6]. In the present study, collagen formation marker PINP was significantly increased during the first 2 years of TNFi. When exploring the net effect of collagen metabolism, the initial balance favored collagen formation as expected [20].

In the process of bone formation, there is consensus that TNF-α mainly acts as inhibitor in the differentiation from pre-osteoblast to osteoblast [21]. Supportive evidence for this theory can be found in our observation of Z-scores below zero from osteoblast product OC at baseline. OC increases after start of TNFi which is in line with previous reports including from our own group [5, 22, 23]. In general, the OC Z-scores remained below zero, indicating a lower bone regulation activity than in the Dutch healthy reference population with similar age and gender. However, 72% and 98% of the OC Z-scores at baseline were between the range of ± 1SD and 2SD, respectively. Movement and weight-bearing exercise are proven to have an osteogenic effect [24]. It is known that due to the disease characteristics in patients with AS especially intensity of exercise is lower than healthy counterparts [25, 26]. Possibly this adds to the presence of negative OC Z-scores. Clinically, these findings are important as it means that TNFi do impact bone homeostasis as reflected by OC levels.

The various roles of TNF-α on disease pathogenesis and its influence on bone formation can also be found in the process of mineralization. TNF-α plays a role in the mineralization process of bone by increasing alkaline phosphatase activity through inhibition of RUNX2 and PPARγ expression [27, 28]. Evidence that this process takes place and leads to observable facilitation of mineralization can be seen in the positive BALP Z-score at baseline. Simultaneously, however, TNF-α can inhibit bone formation by upregulating dickkopf-1 which in turn downregulates wingless pathway signaling [29]. This is part of the TNF brake hypothesis of which the essence is that depending on the inflammatory stage of a lesion bone formation is stimulated or inhibited by TNF-α [30]. In our study, we observed that after start of TNFi facilitator of mineralization marker BALP increases. Mineralization is the last step in the synthesis of new bone, we expected that a significant increase of BALP in comparison to baseline can be seen for a number of years following initiation of TNFi. Previously, we described that BALP was increased during 3 years after start of TNFi [6]. In the present study with 2-year intervals during long-term follow-up, we found that after 2 years levels of BALP decreased compared to previous visits, therefore, also preventing the suggested increased risk in facilitating long-term prolonged mineralization. However, serum levels of BALP remain higher in patients than observed in healthy controls. At 6 years of follow-up, BALP Z-scores above +2SD were seen in 27% of the patients. It is important to monitor these patients in order to identify patients with elevated BALP levels, as they remain at risk for facilitating more excessive mineralization which may also increase fracture risk [31].

A strong aspect of our study is the long-term standardized follow-up including BTM of AS patients with active disease treated with TNFi for 6 years. Following the start of TNFi, disease activity markers ASDAS_CRP_, BASDAI and CRP demonstrated a rapid improvement. In line with previous results, this ensures that anti-inflammatory treatment was given to patients had active disease and was effective, as expected [6]. Furthermore, we calculated BTM Z-scores using a healthy reference group from our hospital to correct for the normal effect of age and gender on BTM course. Furthermore, low serum levels of vitamin D are frequently observed in patient with AS [15]. In comparison, vitamin D levels <50 are also seen in roughly a third of the population in European countries [16]. Inclusion criterion was serum level of 25OHvitD >50 nmol/L for the healthy reference group used to calculate BTM Z-scores. Therefore, it was important to correct for serum levels of vitamin D in the GEE of AS patients. Vitamin D was stable at group level and did not impact the course of BTM when added as potential confounder in the model. Additionally, we eliminated the influence of known fractures and bisphosphonates on BTM.

A potential limitation might be that serum samples were acquired of non-fasting patients (in accordance with daily clinical practice), which may have given more variation in serum levels of BTM. Study visits of the GLAS cohort took place at fixed consultation hour (same half-days), therefore we do not expect a large influence of diurnal variation in our study. Also, the absence of a (historical) reference cohort of untreated AS patients with high disease activity can be seen as limitation. Although short-term data is available from the placebo-controlled trial with infliximab [5], prolonged abstention of TNFi would now be unethical. Therefore, it is not possible to distinguish between the direct and indirect effect of TNFi on the bone metabolism. Furthermore, mSASSS data was only available for a part of the group. However, the course of BTM in these patients and their characteristics were not different from the total study population.

To conclude, after an initial balance favoring collagen formation and facilitation of mineralization, bone turnover markers return to pre-treatment levels during long-term TNF-α inhibition in AS patients. Future research may reveal if BTM can be used as prognostic markers of bone related outcome in AS.

## Abbreviations

25OHvitD: 25-hydroxyvitamin D
AS: ankylosing spondylitis
ASAS: assessments in ankylosing spondylitis
ASDAS_crp_: Ankylosing Spondylitis Disease Activity Score with CRP
BALP: bone-specific alkaline phosphatase
BASDAI: Bath Ankylosing Spondylitis Disease Activity Index
BMD: bone mineral density
BTM: bone turnover marker
CRP: C-reactive protein
ECLIA: electro-chemiluminescence
ELISA: enzyme-linked immunosorbent assay
GEE: generalized estimating equations
GLAS: Groningen-Leeuwarden Axial SpA
HLA-B27: human leukocyte antigen-B27
IBD: inflammatory bowel disease
IE-CV: inter-assay coefficient of variation
IRMA: immunoradiometric assay
mSASSS: modified Stoke ankylosing spondylitis spinal score: OC: osteocalcin
PINP: procollagen type 1 N-terminal peptide
RIA: radioimmunoassay
sCTX: serum type I collagen C-telopeptide
SD: standard deviation
SI: sacroiliac
TNF-α: tumor necrosis factor-alpha
TNFi: TNF-α inhibitor
UMCG: University Medical Center Groningen
